# A comparison of the expected and actual pain experienced by women during insertion of an intrauterine contraceptive device

**DOI:** 10.2147/OAJC.S74624

**Published:** 2015-02-16

**Authors:** Nataliya Brima, Hannat Akintomide, Vivian Iguyovwe, Susan Mann

**Affiliations:** 1 Medical Statistics, Centre for Sexual Health and HIV Research, Research Department of Infection and Population Health, University College London, London, UK; 2 Sexual and Reproductive Health, CNWL Camden Provider Services, Margaret Pyke Centre, London, UK; 3 Department of Sexual and Reproductive Health, Camberwell Sexual Health Centre, Denmark Hill, London, UK; 4 Sexual and Reproductive Health, Kings College Hospital, London, UK

**Keywords:** expected pain, IUD, IUD insertion

## Abstract

**Objective:**

To compare the expected and actual pain experienced with the insertion of intrauterine contraception in women, and to determine whether either of these are related to their personal circumstances, or affected their satisfaction with the procedure.

**Design:**

A convenience sample of 89 women aged 15–50 years attending a sexual health clinic for same day intrauterine contraception insertion were given a questionnaire that they completed following the procedure. The women were asked to rate their expectation of pain prior to insertion and to rate the actual pain they experienced immediately after insertion, on a scale of 1–10, with 10 being severe pain. Information on the women’s circumstances and their level of satisfaction with the procedure was also obtained.

**Results:**

Overall, the median actual pain experienced by women during insertion (4) was significantly lower than the expected pain median (6) (*P*<0.001). For those women who had not had a previous vaginal delivery, actual pain was significantly higher compared with women who had had a previous vaginal delivery (median [interquartile range]: 6 [3.5–7.5] and 3 [1–5], *P*<0.001, respectively), but there was no significant difference between expected and actual pain experiences. In women who had a previous vaginal delivery, actual pain was much lower than expected (*P*<0.001). Neither actual nor expected pain experiences were linked to any other sociodemographic reproductive health or service use factors.

**Conclusion:**

All women had a high expectation of pain prior to IUD insertion, but for those who had had a previous vaginal delivery, this was significantly greater than that actually experienced. Satisfaction levels overall were high. Counseling of women should take into account their expected pain prior to IUD insertion and consideration should be given to alternative and additional methods of pain relief in women who have not had a previous vaginal delivery.

## Introduction

Intrauterine contraception is highly effective at preventing pregnancy. This long-acting reversible contraception (LARC) is cost-effective,^1^ with high continuation and satisfaction rates^2^–^5^ and few contraindications to its use.^6^ It also has added benefits of either being non-hormonal and as effective as emergency contraception (the copper intrauterine device [IUD]) or can be used to reduce menstrual blood loss and endometrial proliferation (the levonorgestrel-impregnated IUD). Despite these benefits, the last decade has seen little change in the proportion of women using intrauterine contraception,^7^ as many eligible women still do not choose this method, perhaps because of their anxiety about pain associated with the insertion procedure.

The scientific literature suggests a continued search for a pharmacological means of reducing pain associated with IUD insertions. Recent studies have investigated misoprostol,^8^–^12^ lidocaine,^13^–^17^ nitroprusside,^18^ and prophylactic ibuprofen,^19^–^21^ and there are plans to use inhaled nitrous oxide (Entonox^®22^). There is little recent research into women’s pain anxieties about the insertion of an IUD.^23^–^26^

The expectation of pain can influence the actual pain women experience during their IUD insertion.^27^ Information and preparation prior to IUD insertion has been shown to reduce anxiety and eventual pain experienced by women during their procedure.^26^ Under current practice women receive counseling but the effect on women’s experiences and satisfaction with intrauterine contraception insertion needs further exploration.

In this study, we sought to determine women’s expected pain and actual pain experienced throughout the procedure, and if either of these was related to sociodemographic factors or reproductive history and whether it influenced their satisfaction with the IUD insertion procedure.

## Design

A convenience sample of 89 women attending the Camberwell Sexual Health Clinic based at King’s College Hospital, London, for same day IUD insertion was included over a 3-month period (between August and October 2011). This clinic provides a walk-in service where IUD insertions are done (where possible on the same day), for emergency contraception, and for ongoing (routine) contraception. Up to 100 IUD insertions are done at the clinic each month. Although all women who were invited to participate agreed to do so, not all eligible women were invited because some clinicians were more likely to recruit patients than others.

Once counseled and having given consent for IUD insertion, the participants were invited to rate how painful they expected the procedure was going to be via a self-completed questionnaire with a numerical scale of 1–10, where 1 represented no pain at all and 10 the most severe pain. Participating women had the insertions of IUDs for both emergency and routine use, including the T-safe^®^ 380A, Nova-T^®^ 380, and Mirena^®^ intrauterine system (IUS) for routine use.

Routine clinic procedure for IUD insertion followed. This included visualization of the cervix, application of tenaculum to the anterior cervical lip, application of 2% lidocaine gel via a quill into the cervical canal, sounding of the uterus with a plastic sound, and insertion of the IUD. A period of 2 minutes was allowed for the lidocaine gel, the local anesthetic, to take effect prior to IUD insertion to comply with the clinic policy. Women for whom the insertion was anticipated to be technically difficult or who preferred paracervical block would be rescheduled in a separate complex contraception clinic. However, none of our study participants required this.

Following IUD insertion and after dressing and being seated, participating women were then asked to self-complete the second part of the questionnaire, about their experience of insertion, including rating the pain they had actually experienced on a similar scale to that described previously. The questionnaire was then returned to but not reviewed by the clinician. Basic sociodemographic factors and reproductive history were recorded for each patient by the clinician using the patients’ clinical records on a separate sheet. The clinician also recorded the type of IUD, circumstance of insertion (routine or emergency), day of cycle, position of uterus, ease of insertion, and their experience in IUD insertion (in terms of number of IUDs ever inserted). Following completion by both the patient and the clinician, the questionnaires were anonymized and were not linked further with the patient notes.

Sample characteristics are presented for all participants. All women in the sample were included in the analysis. To compare pain expectations and experience scores overall and within each women’s delivery history groups, the Wilcoxon test for matched-pairs was used. The relationships between each personal, procedural, and provider’s factors and the actual and expected pain were tested using the chi-square test. All analysis was performed using StataSE 12. A 5% significance level was used.

## Results

A total of 89 women who attended the Camberwell Sexual Health Clinic for same day IUD insertion were included.

The mean age of the cohort was 32 years old (range: 15–50 years). Almost half of the group (49%) had experienced a vaginal delivery in the past and 42% had had an IUD/IUS inserted previously (Table 1).

We analyzed the relationship between each demographic characteristic listed in Table 1 and the level of pain expected and actually experienced (Table 2).

Both actual and expected pains were independent of factors such as age, race, and previous IUD insertion. Actual pain was not related to the type of IUD, reason for insertion (routine or emergency), day of cycle, position of uterus or doctors’ previous experience. We found that actual pain was highly associated with not having experienced a previous vaginal delivery (*P*=0.001; Table 2).

The overall median of expected pain prior to IUD insertion was statistically significantly higher than the overall median of actual pain during IUD insertion (median: 6 and 4, *P*<0.001, respectively; Table 3).

Actual pain was statistically significantly higher for women who had not previously had a vaginal delivery compared to those who had (median: 6 and 3, *P*<0.001, respectively). For women who had not had a previous vaginal delivery, there was no statistically significant difference between expected and actual pain experienced (median: 7 and 6, *P*<0.001, respectively). For those women who had experienced vaginal delivery in the past, actual pain was much lower than expected (median: 3 and 6, *P*<0.001, respectively; Table 3).

Most women (93% of participants) would recommend IUD insertion to a friend. Satisfaction with the procedure of IUD insertion was high – 91% of participants were satisfied with the procedure, awarding a score of 7 or higher on the scale of 1–10.

## Discussion

### Findings of this study and their interpretation

Our findings show that, overall, women in the study experienced less pain during their IUD insertion procedure than they had expected, but this was only statistically significant for women who had previously had a vaginal delivery. For women who had had a previous vaginal delivery, their median pain score was half of what they had expected. Women who had had a previous vaginal delivery therefore appeared to overestimate the potential pain of the procedure while the pain predicted by women who had not had a previous vaginal delivery was in keeping with their actual experience.

There were high numbers of nulliparous and young women in our sample compared to the age distribution of UK community clinic users,^7^ which may be due to the fact that our population was drawn from an integrated sexual health clinic with relatively young patients, that specialist sexual health providers may be more likely to offer and fit IUDs in young nulliparous women, or a changing demand for intrauterine contraception amongst young women.

Despite the pain experienced with IUD insertion, satisfaction levels with the procedure were very high and did not differ for women who did or did not experience severe pain during the insertion.

The vast majority of women would recommend IUD insertion to a friend.

### Strengths and limitations of the study

The data was collected as part of an audit. These findings are limited by the small convenience sample. However, the women were broadly representative of the clinic in terms of their sociodemographic factors. Participation was not limited by potential difficulty of the procedure but some clinicians were more likely to recruit women than others. This may have been related to the experience of the participating clinicians. However, we do not expect this to have influenced the characteristics of the women recruited. We acknowledge that there could be a group of women who did not return to the same clinic for another IUD as a result of their experience, and therefore could bias our sample.

Participants were invited to complete the questionnaire while still in the same room as the clinician. Whilst these women were provided a space to complete this in confidence, it may have influenced the way that the questions were answered. However, we believe using the questionnaire with a numerical scale in our study was more objective than using a visual analog scale (VAS) or a tool that requires administration to the patient by the researcher.

### Relevance of the findings and comparison of the findings with those of other studies

Our study findings confirm evidence available from previous research on pain during IUD insertion: women experience less pain during their IUD insertion procedure than they expect;^27^,^28^ the actual pain experienced by women during the insertion of an IUD is generally low;^21^ and women who have previously had a vaginal delivery are likely to experience little and less severe pain during their procedure than their nulliparous counterparts.^21^,^29^,^30^

In a similar study conducted by Goldstuck and Matthews^27^ in 1985 on nulliparous women, expected pain was significantly higher than actual pain experienced (measured 3 minutes post-insertion), as for our study. Although the devices used in that study, the Copper 7^®^ and Copper 7-mini^®^, are less commonly used in practice today, the inserter diameter is comparable. Of note is that the pain scores obtained in our study were higher than those reported nearly three decades ago, despite use of local anesthesia for our cohort. The studies are both limited by small sample size and sampling techniques and are not directly comparable. However, any increase in pain experienced by women having IUD insertion compared with those three decades ago may reflect a change in pain perceptions or a difference in readiness to report pain and discomfort over time. Difference in the insertion techniques is also likely to be a factor and the use of local anesthetic universally in our study does not appear to have counteracted this effect. As yet, there is no evidence that Instillagel^®^ significantly reduces pain with IUD insertion, although a preliminary randomized study found Instillagel^®^ to be effective for IUD insertion pain if it is allowed at least 5 minutes to take effect.^28^ However, Instillagel^®^ was only allowed 2 minutes to take effect in our study.

As in our study, Carey et al^29^ found pain with IUD insertion in US women to be significantly lower in those who had had a vaginal delivery. Their mean pain scores obtained were also comparable – 3.47 in parous women and 5.12 in nulliparous, though Carey et al^29^ used a different tool (0–10 cm VAS) to measure pain. However, in the present study, there was no insertion difficulty and a greater point difference in pain existed between parous and nulliparous women (3.0 in this study as opposed to 1.51 by Carey et al).^29^

### Recommendations for future research and practice

Participants were not asked nor assessed for anxiety prior to IUD insertion, which may contribute to pain experienced with IUD insertion.^26^,^28^,^31^ However, expected pain has been found to be a significant predictor of pain with IUD insertion,^29^ and may in fact be suggestive of anxiety prior to the procedure. Counseling prior to IUD insertion, including time to answer questions and less formality, discussing what to expect as well as pain-reducing and coping strategies, have been shown to reduce anxiety and pain experienced with procedures.^23^,^24^ All the women in this study were counseled prior to IUD insertion, and irrespective of age, race, parity, or having previously had IUD insertion, still appeared to have a high expectation of pain prior to their procedure, suggesting that women experience high levels of anxiety in relation to IUD insertion.

More research is needed on ways to evaluate pain management strategies for IUD fitting and study the impact of expected pain on the actual experience of fitting. This could include investigating forms of assessment and intervention that may be effective in reducing anxiety prior to insertion that could be incorporated routinely. Given the high level of pain experienced by women who have not had a previous vaginal delivery, consideration should be given to reducing the threshold for local anesthetic use. It would be of interest to ascertain whether the way the information is given by a specific care provider could influence pain perception and reporting.

Future studies should include information such as a psychological assessment of the participants in relation to expected and experienced pain.

## Figures and Tables

**Table 1:** Characteristics of the sample of 89 women^a^

Variables	Groups	n	%
**Sociodemographic factors**			
Age, mean (range)		32 (15–50)	
	≤24 years	18	20
	25–34 years	39	44
	≥35 years	32	36
Ethnicity	White	43	48
	Black	27	33
	Others	19	22
**Reproductive health and service use–related factors**			
Previous vaginal delivery	No	45	51
	Yes	43	49
Previous IUD/IUS insertion	No	52	58
	Yes	37	42
Type of insertion	Emergency	15	18
	Routine	70	82
Type of IUD	Nova-T^®^	19	22
	T-safe^®^	37	42
	IUS	32	36
Position of uterus	Anteverted	61	73
	Retroverted	22	27
Day of cycle	1–5 days	17	25
	6–14 days	32	47
	15–30 days	19	28
Number who have had a previous	<50	19	22
IUD insertion by a clinician	≥50	69	78
**Experiences of pain by patients**			
Expected pain, on a scale of 1–10	1	6	7
	2	5	6
	3	8	9
	4	7	8
	5	13	15
	6	6	7
	7	13	15
	8	12	13
	9	5	5
	10	13	15
Actual pain, on a scale of 1–10	1	14	16
	2	12	14
	3	11	13
	4	8	9
	5	7	8
	6	9	10
	7	13	15
	8	10	11
	9	2	2
	10	2	2

**Note:**

a Total might be less than N=89 due to missing values.

**Abbreviations:** IUD, intrauterine device; IUS, intrauterine system.

**Table 2:** Relationships between sociodemographic, reproductive health, and service use factors; and both expected and experienced pain by women

Variables	Groups	Expected pain, score >5 (n=49)	*P*-value^a^	Actual pain, score >5 (n=36)	*P*-value^a^
		% (n)^b^		% (n)^b^	
**Sociodemographic factors**					
Age	≤24 years	50 (9)	0.813	56 (10)	0.061
	25–34 years	55 (21)		47 (18)	
	≥35 years	59 (19)		25 (8)	
Race	White	59 (25)	0.356	45 (19)	0.613
	Black	44 (12)		33 (9)	
	Others	63 (12)		42 (8)	
**Reproductive health and service use-related factors**					
Previous vaginal delivery	No	61 (27)	0.338	59 (26)	0.001^*^
	Yes	51 (22)		23 (10)	
Previous IUD/IUS insertion	No	55 (28)	0.863	41 (21)	0.952
	Yes	57 (21)		40 (15)	
Type of insertion	Emergency			40 (6)	0.950
	Routine			39 (27)	
Type of IUD	Nova-T^®^			33 (6)	0.641
	T-safe^®^			41 (15)	
	IUS			47 (15)	
Position of uterus	Anteverted			37 (22)	0.275
	Retroverted			50 (11)	
Day of cycle	1–5 days			29 (5)	0.283
	6–14 days			47 (15)	
	15–30 days			56 (10)	
Number who have had previous	<50			33 (6)	0.503
IUD insertion by a clinician	≥50			42 (29)	

**Notes:**

a *P*-value calculated using chi-square test comparing different variables against expected and actually experienced pain (ie, *P-value <0.001 indicates that women who have had a previous vaginal delivery) reported experiencing statistically significantly lower level of pain compared to women who had not had a previous vaginal delivery;

b row percentages given – the corresponding percentages for expected and actual pain score ≤5 have been omitted from the table.

**Abbreviations:** IUD, intrauterine device; IUS, intrauterine system.

**Table 3:** Expected pain vs actual pain by women’s delivery history

Type of pain	No previous vaginal delivery (n=45)	Previous vaginal delivery (n=43)	*P*-value^d^	Total % (n)
Expected pain^a^ Median (IQR)	7 (5–8.5)	6 (3–8)	0.136	6 (4–8)
Actual pain^b^ Median (IQR)	6 (3.5–7.5)	3 (1–5)	<0.001	4 (2–7)
*P*-value^c^	0.083	<0.001		<0.001

**Notes:**

a On a scale of 1–10, how painful do you expect your IUD insertion to be?;

b on a scale of 1–10, what pain did you experience during your IUD insertion?;

c calculated using Wilcoxon matched-pairs signed-rank test;

d calculated using Wilcoxon rank-sum test for unmatched pairs.

**Abbreviation:** IQR, interquartile range.
